# Combining PCR and Metagenomic Approaches to Reveal Tick-Borne Pathogens in Ticks Collected from Livestock and Companion Animals in Cambodia

**DOI:** 10.3390/pathogens15060641

**Published:** 2026-06-16

**Authors:** Sony Yean, Didot Budi Prasetyo, Sovanncheypo Chao, Linavin Vuth, Matthieu Prot, Artem Baidaliuk, Sarah Bonnet, Etienne Simon-Loriere, Sébastien Boyer

**Affiliations:** 1 Medical and Veterinary Entomology Unit, Institut Pasteur du Cambodge, No. 5 Monivong Blvd., P.O. Box 983, Phnom Penh 12201, Cambodia; dbprasetyo@pasteur-kh.org (D.B.P.); csovanncheypo@pasteur-kh.org (S.C.); vlinavin@pasteur-kh.org (L.V.); sboyer@pasteur-kh.org (S.B.); 2 G5 Evolutionary Genomics of RNA Viruses, Pasteur Institute, 75015 Paris, France; matthieu.prot@pasteur.fr (M.P.); artem.baidaliuk@pasteur.fr (A.B.); etienne.simon-loriere@pasteur.fr (E.S.-L.); 3 Ecology and Emergence of Arthropod-Borne Pathogens Unit, Institut Pasteur, Université Paris Cité, CNRS UMR 2000, INRAE USC 1510, 75015 Paris, France; sarah.bonnet@pasteur.fr

**Keywords:** *Rhipicephalus microplus*, *Rhipicephalus linnaei*, tick-borne pathogens, Cambodia

## Abstract

In Cambodia, livestock production plays an important role in the national economy and food security, yet tick-borne diseases remain an underrecognized constraint on animal health and productivity. Domestic animals may also serve as reservoirs of zoonotic pathogens in this predominantly rural setting. To address the lack of baseline molecular data on tick-borne pathogens in Cambodia, we conducted a cross-sectional study of ticks collected from November 2022 to April 2023 across 24 provinces. Ticks were collected from various hosts and environments, including cats, cattle, dogs, goats, pangolins, pythons, wild pigs, and bat cave floors, representing urban, rural, farm, wildlife rescue center, and forest fringe habitats. A total of 1526 ticks belonging to nine species were pooled into 352 samples and screened using conventional PCR (cPCR) targeting *Anaplasma*, *Ehrlichia*, *Babesia*, and *Coxiella*. Additionally, a subset of *Rhipicephalus microplus* ticks was analyzed using metatranscriptomic next-generation sequencing (NGS). *Rhipicephalus microplus* ticks collected from cattle tested positive for *Anaplasma marginale* (1.1% of pools) and *Ehrlichia minasensis* (0.9% of pools), whereas *Rhipicephalus linnaei* ticks collected from dogs were positive for *Anaplasma platys* (0.3% of pools) and *Babesia canis* (2.0% of pools). A high prevalence of Coxiella-like endosymbionts (15.6% of pools) was found in *R. microplus* from both cattle and goats. Metatranscriptomic analysis also identified six tick-associated viruses in *R. microplus* from cattle; with Guangdong tick manly virus being the most dominant (32.5% of samples); followed by Zhangzhou Totiv tick virus 1 (15.0%), Jingmen tick virus (5.0%), and Mogiana tick virus; *Rhipicephalus*-associated rhabdo-like virus; and *Rhipicephalus*-associated flavi-like virus; each at 2.5%. These findings provide the first molecular evidence of numerous bacterial, protozoal, and viral pathogens circulating in *R. microplus* and *R. linnaei* in Cambodia. The study highlights the need for integrated One Health surveillance to better understand, prevent, and control tick-borne diseases in the region.

## 1. Introduction

The public health burden of tick-borne diseases (TBDs) in Cambodia is critically underestimated, and their impact on veterinary health is likely equally significant [1]. Significant gaps in surveillance for both human and animal populations obscure the true diversity, distribution, and impact of tick-associated pathogens [2]. Ticks are major vectors for a wide range of bacterial, viral, and protozoal pathogens, transmitting them among wildlife, livestock, and companion animals, with considerable spillover potential to humans [3,4,5]. Infections from these pathogens can result in severe morbidity and mortality in both human and animal populations, placing a substantial burden on public health and livestock economies [6].

The family *Ixodidae*, commonly known as hard ticks, is part of the order Ixodida and includes some of the most important ectoparasitic vectors affecting both human and domestic hosts [7]. Among these ticks, species from the genus *Rhipicephalus* are particularly significant [8]. The brown dog tick, historically referred to as *Rhipicephalus sanguineus*, is now recognized as a species complex comprising multiple distinct lineages. In tropical Southeast Asia, including Cambodia, the lineage infesting dogs has been identified as *Rhipicephalus linnaei* [9,10]. Pathogens such as *Anaplasma platys*, *Ehrlichia canis*, and *Babesia vogeli* are frequently disseminated to companion animals via *Rhipicephalus linnaei* (the tropical lineage historically categorized within the *R. sanguineus* complex) [6,11,12,13,14]. Concurrently, livestock populations—predominantly cattle, but also including water buffaloes, sheep, and goats—face heavy exposure to *Rhipicephalus microplus*, an ectoparasite of profound economic consequence across the region [15]. Infections caused by *Babesia bovis* and *B. bigemina* have been detected in blood samples from cattle in Thailand [16], Malaysia [17], and Vietnam [18]. Additionally, *Anaplasma marginale* and *Theileria annulata* have been associated with *R. microplus* infestations from cattle in Laos, Thailand, and the Philippines [19,20,21,22]. These pathogens are of major veterinary importance, causing bovine anaplasmosis and theileriosis, respectively, and contributing to significant morbidity and production losses in livestock. In addition, parasites of the genus *Babesia* cause babesiosis in cattle, leading to significant economic losses worldwide ($13.9–$18.7 billion) due to reduced productivity and clinical signs such as anemia, hyperthermia, and hemoglobinuria [16,17,23].

Endemic across multiple Asian territories, Crimean-Congo hemorrhagic fever virus (CCHFV; family *Nairoviridae*, genus *Orthonairovirus*) is fundamentally maintained and transmitted by *Hyalomma* ticks [24,25,26]. CCHFV is mainly transmitted by ticks of the Hyalomma genus, which are the primary vectors and reservoirs for the Crimean-Congo hemorrhagic fever virus [27,28,29,30,31]. While ticks from the *Rhipicephalus* genus may occasionally test positive for viral RNA, this does not necessarily indicate that they are competent vectors [32]. The presence of viral RNA could simply be due to a recent blood meal from an infected host. Moreover, tick-borne encephalitis virus (TBEV), though its role in transmitting these viruses remains uncertain [33], which is spread at least by *Ixodes ricinus* and *Ixodes persulcatus*, is an increasing concern [34]. Another notable pathogen is the Severe fever with thrombocytopenia syndrome virus (SFTSV), belonging to the *Bandavirus* genus (Phenuiviridae family) [35]. This virus has been detected in *Rhipicephalus sanguineus* collected from dogs in Thailand [36], but humans infections are commonly associated with bites from *Haemaphysalis longicornis* [5,37]. In Cambodia, serological evidence shows human exposure to TBEV, CCHFV, SFTSV, Borrelia, Coxiella but active viral circulation in local ticks has not been detected [1]. Overall, ticks play a major role as vectors in the transmission of these viruses, representing a significant threat to both human and animal health [1,25].

Current knowledge of pathogen circulation in Cambodia relies heavily on inferences drawn from regional studies and is limited by a lack of direct, systematic surveillance. Direct molecular detection of pathogens in tick vectors is essential for understanding the prevalence and distribution of these pathogens; however, there is a critical lack of baseline data regarding which pathogens are currently circulating in tick populations infesting domestic and companion animals in Cambodia. To address this knowledge gap, this study aims to establish a comprehensive baseline overview of tick-associated microorganisms in Cambodia using two complementary molecular approaches. We conducted a targeted PCR survey to identify the occurrence and distribution of selected bacterial and protozoan pathogens (specifically *Anaplasma* spp., *Ehrlichia* spp., *Babesia* spp., and *Coxiella* spp.) in ticks collected from cattle, goats, and dogs across 24 provinces. At the same time, we incorporated a metagenomic analysis to provide an initial exploratory assessment of viral diversity within ticks, capturing a broader snapshot of the tick microbiome. While this viromic component was performed on a limited cohort of *Rhipicephalus microplus* ticks as a small-scale pilot study rather than a comprehensive prevalence assessment, its aim was to generate foundational data on tick-associated viruses in Cambodia and evaluate the feasibility of future metagenomic surveillance. Ultimately, by combining these targeted and exploratory molecular approaches, this study seeks to integrate various aspects of the tick microbiome into a unified baseline understanding of Cambodia’s tick-borne microbial diversity.

## 2. Materials and Methods

### 2.1. Ethical Approval

The study was approved and supported by the General Directorate of Animal Health and Production from the Ministry of Agriculture, Forestry, and Fisheries of Cambodia under permit No. 2981, signed on 1 October 2021.

### 2.2. Sample Collection

A cross-sectional tick survey was conducted across 24 provinces in Cambodia between November 2022 to April 2023. Ticks were collected from diverse hosts and environments, including domestic animals, wildlife, and vegetation.

Ticks were randomly selected from domestic and companion animals, specifically cattle, dogs, cats, and goats infested with ticks as determined through visual inspection and full-body palpation. Attached ticks were carefully removed using fine-tipped forceps or tick twisters to preserve specimen integrity. Wildlife-associated ticks were opportunistically collected from pangolins, pythons, and wild pigs. Questing ticks were sampled from vegetation in forest fringe areas using a standard flagging method with a 40 × 60 m white cloth attached to a 1.5 m pole, swiped over taller vegetation like bushes with frequent checks for ticks to prevent them from dropping off, for 30 min [38]. Additionally, bat-associated ticks were collected from cave floors.

All collected specimens were placed in 15 mL conical tubes containing 70% ethanol and labeled with details regarding host species and collection site.

**Figure 1:**
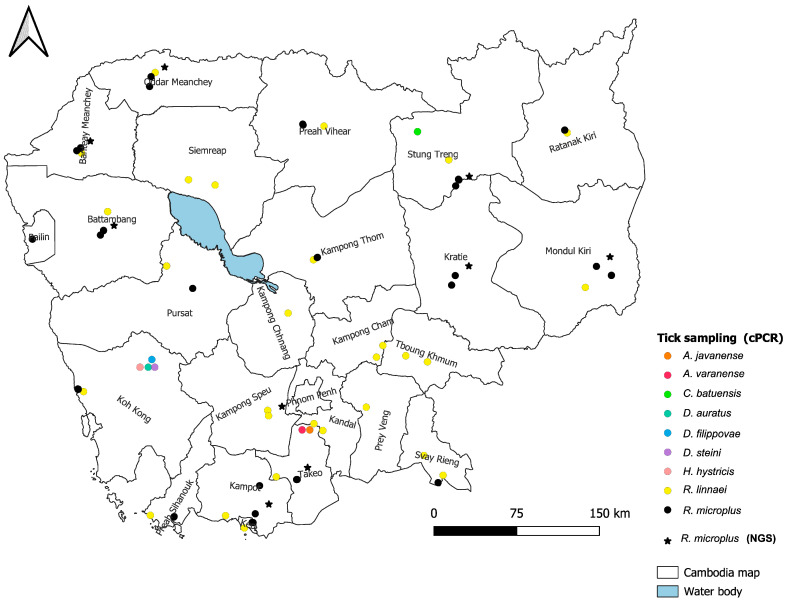
Distribution of tick collection across 24 provinces of Cambodia from cattle, cats, dogs, wildlife, forest-fringe vegetation, and bat cave floors. Samples were screened by cPCR (colored dots), and ticks from 9 provinces were further analyzed using NGS for virus detection (black stars).

For molecular analyses, including conventional polymerase chain reaction (cPCR) and metatranscriptomic next-generation sequencing (mNGS), a subset of 1526 ticks was selected. These specimens were initially identified to the species level using morphological keys developed by the Medical and Veterinary Entomology Unit at the Institut Pasteur du Cambodge (IPC) [39,40,41,42,43,44,45,46]. Ticks were selected from nine species and grouped into 352 pools based on province, species, sex, habitat, and host type, with pool sizes ranging from one to six specimens. For some ticks, a portion was used for taxonomic verification via morphology, while the remaining portion was used for pathogen detection. The distribution of specimens was as follows: *Carios batuensis* (6 pools; 10 females, 10 males, 10 nymphs), *Amblyomma javanense* (2 pools; 1 female, 5 males), *Amblyomma varanense* (2 pools; 5 females, 2 males), Dermacentor auratus (1 specimen, split for molecular identification and pathogen screening), *Dermacentor filippovae* (3 pools; 2 females, 2 males), *Dermacentor steini* (1 specimen, split), *Haemaphysalis hystricis* (1 specimen, split), *Rhipicephalus linnaei* (169 pools; 248 females, 431 males, 3 nymphs), and *Rhipicephalus microplus* (167 pools; 371 females, 417 males, 6 nymphs) (Figure 1).

For viral metagenomic analysis, we randomly selected 40 individual *R. microplus* ticks from the total collection obtained across nine farms (one farm per province, sampling 10 cattle per farm). To achieve balanced geographic and demographic representation, we aimed to select five individual ticks per farm: specifically two adult females, two adult males, and one nymph, where available. Due to lower collection yields in certain areas, fewer specimens were included from Kep (one female, one male, one nymph) and Mondulkiri (two males only). This pilot-scale next-generation sequencing (NGS) approach was designed as a first exploratory investigation of viral diversity in a small set of *R. microplus* ticks collected from cattle in Cambodia. The objective was not to estimate viral prevalence, but we wanted to obtain an initial overview of tick-associated viruses circulating in cattle- associated tick populations and to evaluate the feasibility and usefulness of metagenomic surveillance approaches for future large-scale studies in Cambodia (Figure 1).

### 2.3. Pathogen Screening by Using cPCR

Following a double rinse with distilled water, specimens were grouped into 1.5 mL Eppendorf tubes [47]. Pools of up to 6 ticks were prepared by location, host, species, sex, and developmental stage to increase DNA quantity [48]. To optimize tissue lysis, an abdominal incision was made on each tick prior to overnight incubation at 56 °C in a mixture of 180 µL ATL buffer and 20 µL proteinase K, ensuring complete enzymatic breakdown before supernatant processing [49,50,51]. Cutting open the tick abdomen allowed direct access of the reagents to internal tissues, and overnight incubation ensured thorough enzymatic digestion of the cells, leading to a highly efficient release of high-quality DNA. After transferring 200 µL of the supernatant for the subsequent DNA extraction, the exoskeletons were recovered, washed, preserved in 70% ethanol.

We screened for four pathogens such as *Anaplasma*, *Ehrlichia*, *Babesia*, and *Coxiella* using conventional PCR followed by Sanger sequencing [47]. A 345 bp fragment of the 16S rRNA gene was initially amplified using the EHR16SD/EHR16SR primers for preliminary pathogen screening [52]. Positive samples were subsequently subjected to amplification of an approximately 1500 bp 16S rRNA fragment using the fD1/Rp2 primers to obtain longer sequences for accurate identification and sequencing [53]. *Babesia* was indicated by the band at 207 bp, which represents a fragment of the 18s rRNA gene, using Bcommon-F/Bcommon-R primer sets [54]. *Coxiella* screening targeted a 1322 bp fragment of the 16S rRNA gene using Cox16SF1/Cox16SR2 primer sets [55]. The PCR reactions (40 µL) consisted of 4 µL of 10× buffer B2, 0.8 µL of each primer (10 µM), 0.4 µL of 20 mM dNTPs, 4 µL of 25 mM MgCl_2_, 0.4 µL of Hot FirePol polymerase (5 U/µL), 35 µL of nuclease-free water, and 5 µL of DNA template. To ensure the validity of the PCR assays and monitor for potential contamination, a no-template control (NTC) using nuclease-free water and a positive control consisting of synthetic target DNA fragments (gBlocks™ Gene Fragments, Integrated DNA Technologies, Coralville, IA, USA) were included in every amplification run. Thermal cycling conditions were maintained according to pathogen-specific protocols (Table 1). The PCR products were separated on 1.5% agarose gels and visualized under UV light. Only strong, well-defined bands were sent for DNA sequencing service (Macrogen, Seoul, Republic of Korea).

Sequence data were analyzed using Unipro UGENE v50.0, where raw chromatograms were carefully inspected and cleaned to obtain consensus sequences. The consensus sequences were subjected to BLAST 2.17.0 searches against the NCBI database to validate their preliminary taxonomic identification. Subsequently, sequences were aligned with ClustalW against reference sequences retrieved from GenBank. Phylogenetic trees were constructed using the maximum-likelihood method with 1000 bootstrap replicates in IQ-TREE v2.3.5 [56,57,58]. The resulting trees were visualized and edited in MEGA v11.0.13 [50,59] (Table S1).

### 2.4. Pathogen Screening by Using Next Generation Sequencing

#### 2.4.1. RNA Extraction and Library Preparation

Individual ticks were homogenized, and RNA was extracted using the Qiagen QIAamp Viral RNA Kit (QIAGEN, Hilden, Germany) with minor modifications including extended tissue homogenization and lysis steps to optimize yield and reduce RNA degradation. RNA quality and concentration were measured using a Qubit fluorometer (Thermo Fisher Scientific, Waltham, MA, USA). For library preparation, the extracted RNA was treated with DNase, depleted of ribosomal RNA, and reverse-transcribed to cDNA before library preparation using a Nextera XT kit (Illumina, San Diego, CA, USA). Sequencing was performed using NextSeq 500/550 v2.5 kit (Illumina, San Diego, CA, USA).

#### 2.4.2. Bioinformatic Analysis

Reads were demultiplexed with bcl2fastq v2.2 and quality-checked using FastQC v0.11.9. Reads were quality-trimmed with trimmomatic v0.39 and de novo assembled using MEGAHIT v1.2.9. Viral sequences were identified through BLASTx/BLASTn searches against the NCBI non-redundant protein database [60]. Reads were mapped to reference viral genomes using Bowtie2 v2.1.0 to quantify abundance. The number of mapped reads was normalized as reads per million (RPM) to account for differences in sequencing depth among samples. The newly generated sequences have been submitted to SRA under Bioproject number PRJNA1476874.

### 2.5. Statistical Analysis

Pathogen prevalence was expressed as the percentage of positive tick pools relative to the total number of pools tested for each pathogen, host, or tick species [61,62]. The Minimum Infection Rate (MIR) was estimated assuming one infected tick per positive pool and calculated as the number of positive pools divided by the total number of ticks tested at each site. MIRs were also calculated separately by tick developmental stage and sex (adults, nymphs, females) to account for variation in infection rates among *Anaplasma* sp., *Babesia* sp., *Ehrlichia* sp., and *Coxiella* sp. [63,64].

## 3. Results

### 3.1. PCR Results

A total of 352 tick pools were screened using PCR for *Anaplasma* sp., *Babesia* sp., *Ehrlichia* sp., and *Coxiella* sp. Among these, 135 pools tested positive for at least one of the pathogens. In the positive tick pools, single-pathogen detections were the most common, comprising 85.93% (*n* = 116) of the samples. Dual-pathogen detections accounted for 13.33% (*n* = 18) of the pools, while triple-pathogen detections indicating the presence of three different pathogens in a single pooled sample were quite rare, occurring in only 0.74% (*n* = 1) of the pools. Overall, a total of 155 pathogen detections were recorded, which shows that the co-detection of multiple pathogens occurred in some tick pools. Of these detections, 105 PCR amplicons were successfully sequenced; however, 50 amplicons could not be sequenced due to faint PCR bands that were insufficient for reliable sequencing. Sequencing success was achieved across all infection categories, including the sequencing of all detected pathogens in the triple-infected pool (Table 2). The composition of the tick pools varied by species and host, which have influenced the detection of pathogens. Specifically, the pools included the following: *C. batuensis* collected from bat cave floors (*n* = 6), *R. linnaei* from cats (*n* = 4) and dogs (*n* = 161), as well as from cattle (*n* = 4), *R. microplus* from cattle (*n* = 162), dogs (*n* = 3), and goats (*n* = 2), *A. javanense* from pangolins (*Manis javanica*) (*n* = 2), *A. varanense* from pythons (*Malayopython reticulatus*) (*n* = 2), half-individual sample of *D. steini* (*n* = 0.5), and *H. hystricis* (*n* = 0.5) collected from vegetation, half-individual sample of *D. auratus* from wild pigs (*Sus scrofa*) (*n* = 0.5) and *D. filippovae* from wild pigs (*n* = 3) (Table 3).

Overall, pathogen screening revealed a total pool positivity rate of 44.0% (155/352) across the entire study. When broken down by specific host–tick combinations out of the total sample size (*n* = 352), *Rhipicephalus microplus* pools from cattle accounted for the largest share of pathogen detections at 33.8% (119/352), followed by *Rhipicephalus linnaei* pools from dogs at 9.3% (33/352), *R. microplus* pools from goats at 0.6% (2/352), and *Carios batuensis* pools from bat cave floors at 0.3% (1/352). Looking at individual pathogen targets across the entire study, *Coxiella-like endosymbionts* were detected in 15.6% (55/352) of all pools, followed by *Anaplasma* spp. at 13.3% (47/352), *Coxiella* spp. at 8.2% (29/352), and *Babesia* spp. at 2.0% (7/352). Lower overall prevalence rates were observed for *Anaplasma marginale* at 1.1% (4/352), *Ehrlichia* spp. at 1.7% (6/352), *Babesia canis* at 0.3% (1/352), *Anaplasma platys* at 0.3% (1/352), and *Ehrlichia minasensis* at 0.9% (3/352). Finally, tick pools recovered from cats (0/352), pangolins (0/352), pythons (0/352), vegetation (0/352), and wild pigs (0/352) yielded no detectable bacterial or parasitic DNA (Table 3A).

The minimum infection rate (MIR) was calculated to provide a conservative estimate, though it typically underestimates the true prevalence of tick-borne pathogens. A total of 1526 individual ticks were examined for tick-borne pathogens. Overall, 753 ticks (49.4% MIR) were positive for at least one pathogen. *Coxiella*-like endosymbionts (CLE) were the most frequently detected agents, identified in 275 ticks (18.1%), followed by *Anaplasma* spp. in 238 ticks (15.6%) and *Coxiella* spp. in 123 ticks (8.1%). *Ehrlichia minasensis* and *Ehrlichia* spp. were detected in 18 (1.2%) and 34 (2.2%) ticks, respectively. Among protozoan pathogens, *Babesia canis* was detected in 30 ticks (2.0%), while *Babesia* spp. were identified in 14 ticks (0.9%). Most positive detections were observed in *Rhipicephalus microplus* collected from cattle, whereas *Rhipicephalus linnaei* collected from dogs showed detection of *Anaplasma* spp., *A. platys*, and *Babesia canis*. Additionally, five ticks collected from bat cave floors and identified as *Carios batuensis* were positive for *Anaplasma* spp. (0.3% MIR) (Table 3B).

### 3.2. DNA Sequencing and Phylogenetic Analysis

**Figure 2:**
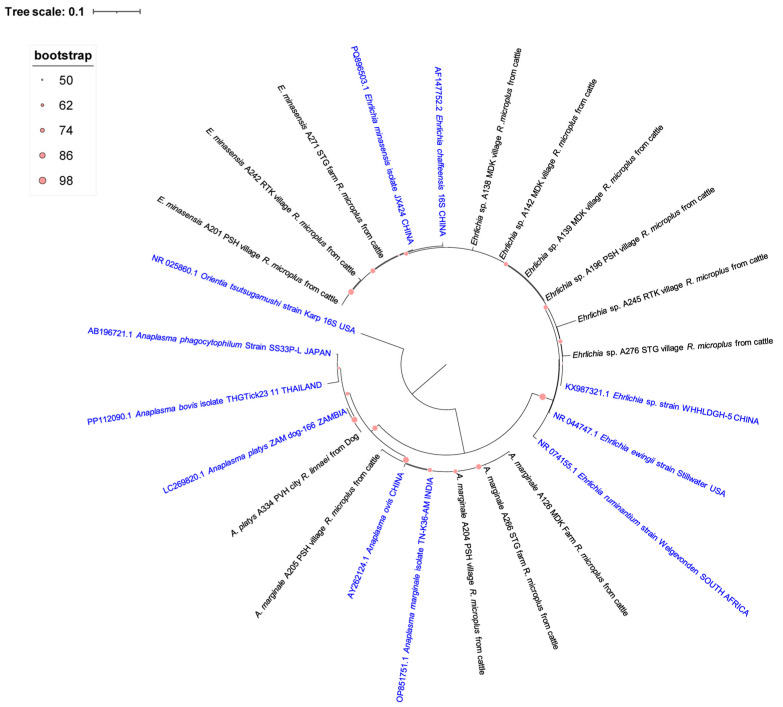
A maximum-likelihood phylogenetic tree was constructed using 16S rRNA gene sequences to explain the relationships between *Anaplasma* and *Ehrlichia* species detected in *Rhipicephalus microplus* and *R. linnaei* ticks on cattle and dog across different region in Cambodia, alongside reference sequences from GenBank under accession number (PZ515790-PZ515794), (PZ537012-PZ537020). The tree was rooted using *Orientia tsutsugamushi* as an outgroup. The sequences obtained from this study are highlighted in black.

A maximum-likelihood phylogenetic tree based on 16S rRNA gene sequences revealed genetic relatedness among *Anaplasma* and *Ehrlichia* isolates from *Rhipicephalus microplus* and *R. linnaei* ticks infesting cattle and dogs in Cambodia (Figure 1). *Anaplasma marginale* strains from Preah Sihanouk (PSH), Mondulkiri (MDK), and Stung Treng (STG) showed >99% similarity to an Indian reference strain (OP851751.1). *Anaplasma platys* was detected in a pool of *R. linnaei* collected from a dog in Preah Vihear (PVH) and shared 99.68% similarity with a Zambian strain (LC269820.1). *Ehrlichia minasensis* isolates from cattle in Preah Sihanouk, Rattanakiri, and Stung Treng exhibited >99% similarity to a Chinese strain (PQ896500.1). An unidentified *Ehrlichia* sp. displayed 96.11–99.84% similarity to the closest match *Ehrlichia* sp. in Chinese (KX987321.1) (Figure 2).

**Figure 3:**
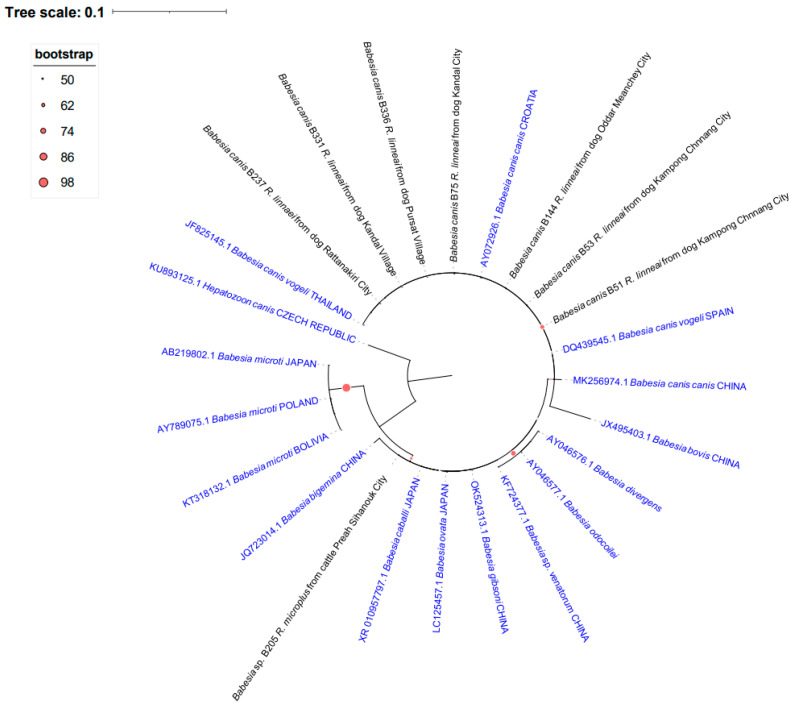
A maximum-likelihood phylogenetic tree was constructed using 18S rRNA gene sequences to visualize the relationships between *Babesia canis* and *Babesia* sp. detected in *Rhipicephalus linnaei* and *R. microplus* ticks on dogs and cattle across different regions in Cambodia, alongside reference sequences from GenBank under accession number (PZ509201-PZ509194). The tree was rooted using *Hepatozoon canis* as an appropriate outgroup. The sequences obtained from this study are highlighted in blue.

Phylogenetic analysis of 18S rRNA sequences observed that *Babesia canis* strains from the *R. linnaei* pool were found on dogs in Ratanakiri, Kandal, Pursat, Odar Meanchey, and Kampong Chhnang. Sequence analysis confirmed that these isolates belonged to the *Babesia canis* complex. However, the genetic marker used in this study did not provide sufficient resolution to distinguish among the closely related subspecies (*B. canis canis*, or *B. canis vogeli*). Given that *R. linnaei* is the predominant tick species parasitizing dogs in the region and is a recognized vector of *B. canis vogeli* in Asia, the findings are epidemiologically consistent with this subspecies, although definitive subspecies identification could not be established. These results indicate the circulation of *B. canis* (sensu lato) among companion animals and associated ticks in the surveyed areas. In contrast, a *Babesia* sp. detected in *R. microplus* from cattle in Preah Sihanouk showed no close relationship to any known sequences, indicating a distinct lineage (Figure 3).

**Figure 4:**
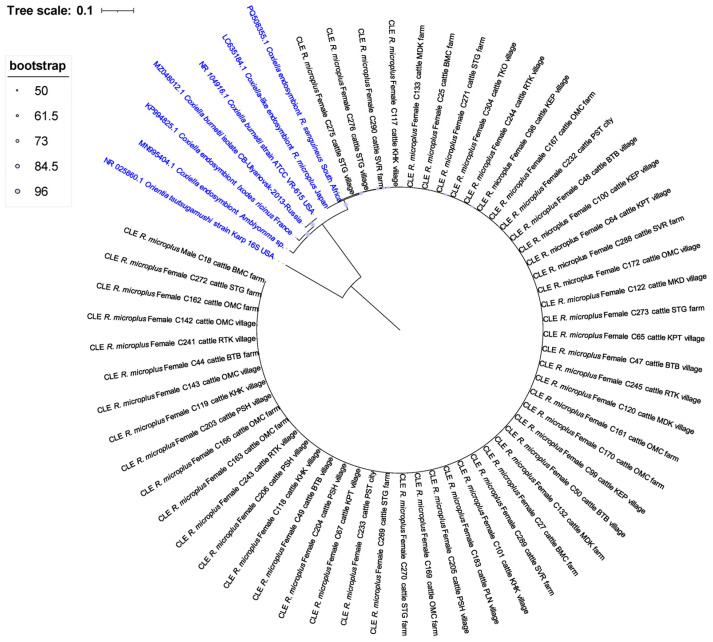
A maximum-likelihood phylogenetic tree was constructed using 16S rRNA gene sequences to visualize the relationships between Coxiella-like endosymbionts detected in *Rhipicephalus microplus* ticks on cattle across different regions in Cambodia, alongside reference sequences from GenBank under accession numbers (PZ510385-PZ510439). The tree was rooted using *Orientia tsutsugamushi* as an appropriate outgroup. The sequences obtained from this study are highlighted in black.

All 55 *Coxiella*-like endosymbiont sequences obtained from *R. microplus* ticks on cattle across 14 Cambodian provinces including Banteay Meanchey, Battambang, Kampong Thom, Kep, Koh Kong, Mondulkiri, Oddar Meanchey, Pailin, Preah Sihanouk, Pursat, Ratanakiri, Stung Treng, Svay Rieng, and Takeo exhibited >99% similarity to a Coxiella endosymbiont of *Rhipicephalus sanguineus* from South Africa (PQ508355.1) (Figure 4).

### 3.3. Virus Detection in Rhipicephalus microplus

Analysis of 40 *Rhipicephalus microplus* ticks processed through mNGS revealed the detection of viral RNA in 18 samples (45%), while 22 samples (55%) were negative under the analytical parameters used. Six distinct tick-associated viruses were identified: Guangdong tick manly virus (GTMV), Jingmen tick virus (JMTV), Mogiana tick virus (MGTV), *Rhipicephalus*-associated rhabdo-like virus, *Rhipicephalus*-associated flavi-like virus, and Zhangzhou totiv tick virus 1. Single-virus infections were detected in 14 ticks (35%), dual infections in 3 ticks (7.5%), and co-infection with four viruses in 1 tick (2.5%), indicating heterogeneous viral burdens among individual ticks. GTMV was the most prevalent virus, detected in 12 samples (32.5%) from Battambang, Oddar Meanchey, Takeo, and Kampong Speu provinces, predominantly in male ticks (7 males, 2 females, 3 nymphs). JMTV was identified in two samples (5%) from Stung Treng and Mondulkiri (one male, one female). Mogiana tick virus, *Rhipicephalus*-associated rhabdo-like virus, and *Rhipicephalus*-associated flavi-like virus were each detected in single samples (2.5%), primarily from Mondulkiri and Kampong Speu provinces. Zhangzhou totiv tick virus 1 was detected in six samples (15%) across five provinces, mostly in male ticks (Table 4).

### 3.4. Viral Abundance and Genome Coverage

Metatranscriptomic sequencing generated 258,910,074 total reads, with an average of 20.8 million reads per sample. GTMV exhibited the highest abundance and genomic completeness. One male tick from Battambang showed a high viral load, with GTMV reads comprising 20.6% of total RNA (~205,800 read per million). Across all GTMV-positive samples, read counts ranged from 12,531 to 360,367, with 99–100% genome coverage and ~96% nucleotide identity to the reference genome (OM264164.1). Zhangzhou totiv tick virus 1 showed read counts ranging from 1880 to 9360 RPM, with 98–100% genome coverage and 94–96% nucleotide identity. JMTV ranged from 1403 to 216,265 reads with genome coverage of 90–97% and ~93% identity, with read abundances up to 217,668 RPM. MGTV and both *Rhipicephalus*-associated rhabdo-like and flavi-like viruses displayed near-complete to complete genome coverage (97–100%) and nucleotide identities ranging from 93 to 95% relative to reference genomes (MT080097.1, ON812505.1, MH814979.1).

## 4. Discussion

Our study provides new molecular evidence regarding the circulation of tick-borne pathogens in *Rhipicephalus microplus* and *R. linnaei* collected from cattle, goats, and dogs throughout Cambodia. During field sampling, adult ticks were collected in greater numbers than nymphs and larvae. This discrepancy is primarily attributed to the larger size and higher visibility of adult stages, whereas immature stages—particularly larvae—are considerably smaller, more difficult to detect under field conditions, and heavily constrained by collection time limits. Despite these sampling limitations, we detected two bacterial agents: *Anaplasma marginale*, and *Ehrlichia minasensis*, as well as non-pathogenic Coxiella-like endosymbionts. Additionally, we found the protozoan pathogen *Babesia canis* and several tick-associated viruses, including the JMTV, which is known to infect both animals and humans [65,66,67,68,69,70,71].

*Anaplasma marginale* primarily infects cattle and is not known to cause human infection, in contrast to other members of the genus such as *Anaplasma phagocytophilum* which are zoonotic [6,14,72,73,74]. The prevalence of *A. marginale* in Cambodian *R. microplus* (1.1%) was similar to Vietnam data (1%), but it was slightly lower than reported in the Philippines (6.7%), and Thailand (19.08%) [6,72,75].

The detection of *E. minasensis* is particularly noteworthy; this emerging *Ehrlichia* species has close phylogenetic relationships with recognized human pathogens and has recently been identified in cattle ticks globally [14,72,76,77]. In our study, *E. minasensis* was detected in 0.9% of Cambodian *R. microplus*. This prevalence is comparable to low levels reported in Thailand (1.97%) [72].

Moreover, *Anaplasma platys* is a canine pathogen causing cyclic thrombocytopenia; it was detected in 0.3% of *R. linnaei*. This prevalence is lower than reports from Thailand (22.4%) [78]. We also detected *Babesia canis*, a protozoan agent of canine babesiosis, confirmed to species level by 18S rRNA sequencing [11]. Reports of *B. canis* in *R. linnaei* in Cambodia are limited, with a prevalence of 2.0% observed in our study.

In contrast, the *Babesia* sp. detected within *R. microplus* ticks collected from cattle in Preah Sihanouk presents a highly remarkable and unexpected finding. Downstream sequence verification demonstrated that this isolate shared no high-confidence identity or homology with any well-characterized *Babesia* references currently available in the NCBI GenBank database. This sharp divergence strongly indicates the presence of an unmapped, distinct, or novel *Babesia* lineage circulating locally in southern Cambodia. However, interpretation of this result should be made cautiously because the sequence quality was limited, likely due to low DNA concentration and faint PCR amplification, which may reduce the accuracy of BLAST identification and phylogenetic inference. In addition, partial 18S rRNA sequences may not always provide sufficient resolution for reliable species-level discrimination among closely related piroplasms [35,48]. Although *Coxiella burnetii* was not detected in our *R. microplus* ticks, we observed a high prevalence of CLEs. CLEs are closely related to *C. burnetiid*, the etiologic agent of Q fever in humans and coxiellosis in animals [79,80]. CLE are increasingly recognized as important components of tick biology, influencing reproduction, survival, and potentially vector competence for other pathogens [19,81]. The absence of *C. burnetii* may reflect its low prevalence in Cambodia compared to data reported from the Philippines (10.2%) in *R. microplus* [75]. The non-detection of *Coxiella* sp. is likely due to the limited sample size, single time-point collection, and the restricted host range sampled (cattle, cats, dogs, goats, and few wildlife animals), which may have reduced the probability of detecting the prevalence of pathogens.

In addition to bacterial and protozoal agents, metagenomic analysis of *R. microplus* has identified six tick-associated viruses, among which JMTV is notable [69,70,82]. JMTV has been detected in both human febrile cases and animal hosts in various settings across Asia [67]. The presence of JMTV in *R. microplus* specimens collected from cattle farms in Cambodia indicates a geographic expansion of the virus which has primarily been reported in China, suggesting it may now be circulating more widely throughout Southeast Asia. Because *R. microplus* commonly infests cattle and will occasionally bite humans, this detection raises important public health and veterinary concerns [83]. In humans, JMTV infection is typically associated with nonspecific febrile illness, including fever, headache, myalgia, malaise, and occasionally gastrointestinal symptoms or rash. In livestock, *R. microplus* is already a major pest responsible for substantial economic losses; the presence of a viral pathogen further amplifies its significance in veterinary medicine, even though clinical signs of JMTV in animals remain poorly documented. These risks are compounded by the local context in Cambodia, where tick-borne viral infections are likely underdiagnosed and underreported. Due to limited clinical awareness and diagnostic capacity, veterinarians and physicians may not readily recognize tick-borne diseases. Because clinical signs in livestock are often nonspecific such as fever, lethargy, reduced appetite, and decreased productivity, they are easily misdiagnosed or attributed to other endemic conditions, and some infected animals may remain asymptomatic, further obscuring clinical identification. However, the findings of this study must be interpreted cautiously. The detection of JMTV RNA in Cambodian *R. microplus* confirms its circulation within the tick population, but RNA presence alone does not confirm active viral replication, vector competence, or successful transmission to livestock and humans. While JMTV is linked to human illness in other regions, its clinical significance in Cambodia remains unknown, and no direct evidence of human or animal disease linked to JMTV was obtained in this study. Therefore, conclusions regarding zoonotic risk should remain tentative, and future investigations including virus isolation, transmission dynamics studies, and serological surveys are required to determine its true epidemiological relevance and to sustain vital tick surveillance in the region.

Several limitations should be acknowledged. First, the cross-sectional design does not capture temporal dynamics of tick activity and pathogen circulation. Second, while sampling spanned multiple provinces, localized pathogen diversity may remain underrepresented. Third, the absence of parallel serological or clinical surveillance in animals and humans limits our ability to confirm exposure history or disease associations [84]. Finally, conventional PCR, while specific, can give false positive or false negative results [85], while the use of pooled tick samples restricts the calculation of exact individual prevalence. Furthermore, reliance on reference database–dependent sequence classification introduces potential uncertainty and bias in precise taxonomic assignment, particularly for less-characterized variants.

## 5. Conclusions

The study provides foundational molecular evidence that tick-borne pathogens, which are significant for veterinary and potential zoonotic concerns, are present in Cambodia. To better understand transmission pathways, assess the economic impact on livestock, and evaluate potential public health risks, it is crucial to expand integrated “One Health” surveillance. This approach should incorporate tick identification, pathogen detection, and host-level diagnostics. Future research that combines longitudinal entomological sampling, host serology, and clinical diagnostics will yield a more comprehensive understanding of the ecology of tick-borne diseases in Cambodia.

## Figures and Tables

**Table 1:** List of TBPs, target genes, PCR primer name and PCR program in TBP screening.

Target	Gene	Primer Name	Sequence (5′ to 3′)	Product Size	PCR Program	References
*Anaplasmatacae*	16S rRNA	EHR16SD EHR16SR	5′-GGTACCYACAGAAGAAGTCC-3′ 5′-TAGGACTCATCGTTTACAGC-3′	345 bp	Denaturation 95 °C 15 min, 94 °C 1 min, annealing 58 °C 30 s, Extension 72 °C 30 s and final extension 72 °C 5 min (40 cycles), and hold 4 °C	[52]
		fD1 Rp2	5′-AGAGTTTGATCCTGGCTCAG-3′ 5′-ACGGCTACCTTGTTACGACTT-3′	1500 bp	Denaturation 95 °C 15 min, 94 °C 1 min, annealing 58 °C 30 s, Extension 72 °C 30 s and final extension 72 °C 5 min (40 cycles), and hold 4 °C	[53]
*Babesia* spp.	18S rRNA	Bcommon-F Bcommon-R	5′-GCATTTGCGATGGACCATTCAAG-3′ 5′-CCTGTATTGTTATTTCTTGTCACTACCTC-3′	207 bp	Denaturation 95 °C 15 min, 94 °C 30 s, annealing 58 °C 30 s, Extension 72 °C 30 s and final extension72 °C 5 min (40 cycles), and hold 4 °C	[54]
*Coxiella* spp.	16S rRNA	Cox16SF1 Cox16SR2	5′-CGTAGGAATCTACCTTRTAGWGG-3′ 5′-GCCTACCCGCTTCTGGTACAATT-3′	1322 bp	Denaturation 95 °C 15 min, 93 °C 30 s, annealing 60 °C 30 s, Extension 72 °C 1 min and final extension 72 °C 5 min (40 cycles), and hold 4 °C	[55]

**Table 2:** Distribution of infection status and sequencing outcomes among PCR-positive tick pools for Anaplasmatacea, *Coxiella* spp., and *Babesia* spp.

Infection Status	Number of Pools	% of Positive Pools	Pathogen Detections	Sequenced	Not Sequenced (Faint Band)
Single Infection	116	85.93%	116	78	38
Dual infection	18	13.33%	36	24	12
Triple infection	1	0.74%	3	3	0
Total	135	100%	155	105	50

**Table 3:** (**A**) Prevalence of tick samples collected in 24 provinces that harbored DNA of selected genera of bacteria and parasites according to tick species and host species. (**B**) Minimum infection rate (MIR) of tick-borne pathogens across different host species per tick species.

(**A**)												
**Host**	**Species**	**No. of Tick Pool**	**Anaplamosis**	***A. marginale***	***A. platys***	***B. canis***	***B.*** **spp.**	**CLE**	***C.*** **spp.**	***E. minasensis***	***E.*** **spp.**	**Total**
Bat cave floor	*C. batuensis*	6	1 (0.3)	-	-	-	-	-	-	-	-	1 (0.3)
Cattle	*R. linnaei*	4	-	-	-	-	-	-	-	-	-	0
	*R. microplus*	162	23 (6.5)	4 (1.1)	-	-	1 (0.3)	54 (15.3)	28 (7.9)	3 (0.9)	6 (1.7)	119 (33.8)
Dog	*R. linnaei*	161	23 (6.5)	-	1 (0.3)	7 (2.0)	2 (0.6)	-	-	-	-	33 (9.3)
	*R. microplus*	3	-	-	-	-	-	-	-	-	-	0
Goat	*R. microplus*	2	-	-	-	-	-	1 (0.3)	1 (0.3)	-	-	2 (0.6)
	Grand Total/(% of positive pools)	352	47 (13.3)	4 (1.1)	1 (0.3)	7 (2.0)	3 (0.9)	55 (15.6)	29 (8.2)	3 (0.9)	6 (1.7)	155 (44.0)
(**B**)												
**Host**	**Species**	**No. of Tick Pool**	**Anaplamosis**	***A. marginale***	***A. platys***	***B. canis***	***B.*** **spp.**	**CLE**	***C.*** **spp.**	***E. minasensis***	***E.*** **spp.**	**Total**
Bat cave floor	*C. batuensis*	30	5 (0.3)	-	-	-	-	-	-	-	-	5 (0.3)
Cattle	*R. linnaei*	16	-	-	-	-	-	-	-	-	-	0
	*R. microplus*	782	118 (7.7)	16 (1.1)	-	-	5 (0.3)	274 (18.0)	119 (7.8)	18 (1.2)	34 (2.2)	584 (38.3)
Dog	*R. linnaei*	649	115 (7.5)	-	4 (0.3)	30 (2.0)	9 (0.6)	-	-	-	-	158 (10.4)
	*R. microplus*	6	-	-	-	-	-	-	-	-	-	0
Goat	*R. microplus*	6	-	-	-	-	-	1 (0.1)	5 (0.3)	-	-	6 (0.4)
	Grand Total number of individual tick/(MIR %)	1526	238 (15.6)	16 (1.1)	4 (0.3)	30 (2.0)	14 (0.9)	275 (18.1)	123 (8.1)	18 (1.2)	34 (2.2)	753 (49.4)

Abbreviations: *Amblyomma* (A.), *Carios* (C.), *Dermacentor* (D.), *Haemaphysalis* (H.), *Rhipicephalus* (R.), *Anaplasma* (A.), *Babesia* (B.), *Coxiella* (C.), *Ehrlichia* (E.), and Coxiella-like endosymbiont (CLE).

**Table 4:** Summary of viruses detected from *Rhipicephalus microplus*.

Virus Name	Positive Samples (*n*)	Province (s)	Tick Sex	Zoonotic	Vector	Distribution	References
Guangdong tick manly virus	12	Battambang	2 male, 2 female	Unknown	*Rhipicephalus* spp.	China	[65]
		Oddar Meanchey	2 male, 1 nymph				
		Kampong Speu	2 male, 1 nymph				
		Takeo	1 male, 1 nymph				
Jingmen tick virus	2	Stung Treng	1 female	Animal & Human [66]	*R. microplus*, *H. longicornis*, *H. campanulata*, *I. granulatus*, *H. flava*, *I. sinensis*, *Bos* sp., *R. sanguineus*, *Armigeres* sp.	China, Asia, Americas, Europe	[66,67]
		Mondulkiri	1 male				
Mogiana tick virus	1	Mondulkiri	1 male	Unknown	*R. microplus*	Brazil	[68]
Rhipicephalus associated rhabdo-like virus	1	Kampong Speu	1 female	Unknown	*R. microplus*	China	[66,69]
Rhipicephalus associated flavi-like virus	1	Mondulkiri	1 male	Unknown	*R. microplus*	China	[69]
Zhangzhou Totiv tick virus 1	6	Oddar Meanchey	1 male	Unknown	Unknown	Unknown	*NCBI: txid2972350*
		Banteay Meanchey	1 female				
		Kep	1 male				
		Takeo	1 male				
		Kampong Speu	1 male, 1 female				

## Data Availability

The newly generated sequences have been submitted to SRA under Bioproject number PRJNA1476874.

## Supplementary Materials

The following supporting information can be downloaded at: https://www.mdpi.com/article/10.3390/pathogens15060641/s1, Table S1: BLASTn results of sequenced PCR amplicons obtained from tick samples collected in Cambodia.
