# Supplementary material for: Combining PCR and Metagenomic Approaches to Reveal Tick-Borne Pathogens in Ticks Collected from Livestock and Companion Animals in Cambodia
Source: Pathogens. 2026 Jun 16;15(6):641. doi: 10.3390/pathogens15060641 (PMC13304860; doi:10.3390/pathogens15060641)
Supplement: Supplementary file 1 [file pathogens-15-00641-s001.zip › pathogens-4269100-Supplementary.pdf]

Supplementary Table S1. BLASTn results of sequenced PCR amplicons obtained from tick samples collected in Cambodia. The table includes sample identification, target pathogen, closest GenBank match, accession number, query coverage, percentage identity, and E-value used for taxonomic assignment.

Accesion No.	Sample ID	Pathogen Detected	Target Gene	Sequence Length (bp)	Closest Match (BLASTn)	GenBank Accession No.	Query Cover (%)	Identity (%)	E-value	Country of Reference Sequence
PZ515790	A126	*Anaplasma marginale*	16S rRNA	617	*Anaplasma marginale* isolate F57	OL660542.1	100	99.68	0	Taiwan
PZ537012	A138	*Ehrlichia* sp.	16S rRNA	617	Uncultured *Ehrlichia* sp. clone CRETAV-HUVDH	OK481113.1	100	99.35	0	Angola
PZ537013	A139	*Ehrlichia* sp.	16S rRNA	617	Uncultured *Ehrlichia* sp. clone CRETAV-HUVDH	OK481113.1	100	100	0	Angola
PZ537014	A142	*Ehrlichia* sp.	16S rRNA	617	*Ehrlichia* sp. strain WHBMXZ-42-1	KX987323.1	100	100	0	China
PZ537015	A196	*Ehrlichia* sp.	16S rRNA	617	Uncultured *Ehrlichia* sp. clone CRETAV-HUVDH	OK481113.1	100	100	0	Angola
PZ537018	A201	*Ehrlichia minasensis*	16S rRNA	617	*Ehrlichia minasensis* isolate E-2650	MH500005.1	100	99.84	0	Australia
PZ515791	A204	*Anaplasma marginale*	16S rRNA	617	*Anaplasma marginale* isolate F57	OL660542.1	100	99.68	0	Taiwan
PZ515792	A205	*Anaplasma marginale*	16S rRNA	617	*Anaplasma marginale* isolate ZJ02/2009	HM439433.1	100	99.19	0	China
PZ537019	A242	*Ehrlichia minasensis*	16S rRNA	617	*Ehrlichia minasensis* isolate E-2650	MH500005.1	100	99.19	0	Australia
PZ537016	A245	*Ehrlichia* sp.	16S rRNA	617	Uncultured *Ehrlichia* sp. clone MieHl173	MT258398.1	100	96.11	0	Japan
PZ515793	A266	*Anaplasma marginale*	16S rRNA	617	*Anaplasma marginale* isolate F57	OL660542.1	100	99.68	0	Taiwan
PZ537020	A271	*Ehrlichia minasensis*	16S rRNA	617	Uncultured *Ehrlichia* sp. clone HbYS117	OR508727.1	100	100	0	China
PZ537017	A276	*Ehrlichia* sp.	16S rRNA	617	Uncultured *Ehrlichia* sp. clone MieHl173	MT258398.1	100	98.38	0	Japan
PZ515794	A334	*Anaplasma platys*	16S rRNA	617	*Anaplasma platys* strain S3	CP046391.1	100	99.68	0	Saint Kitts and Nevis
PZ509201	B336	*Babesia canis*	18S rRNA	166	*Babesia canis* isolate 3569	MW939359.1	100	100	3.00E-79	Romania
PZ509200	B331	*Babesia canis*	18S rRNA	166	*Babesia canis* isolate 3569	MW939359.1	100	100	3.00E-79	Romania
PZ509199	B237	*Babesia canis*	18S rRNA	166	*Babesia canis* isolate 3569	MW939359.1	100	100	3.00E-79	Romania
PZ509198	B205	*Babesia* sp.	18S rRNA	166	*Babesia bigemina* isolate TKX-1	PV202466.1	99	100	4.00E-78	China
PZ509197	B144	*Babesia canis*	18S rRNA	166	*Babesia canis* isolate 3569	MW939359.1	100	100	3.00E-79	Romania
PZ509196	B75	*Babesia canis*	18S rRNA	166	*Babesia canis* isolate 3569	MW939359.1	100	100	3.00E-79	Romania
PZ509195	B53	*Babesia canis*	18S rRNA	166	*Babesia cani* s isolate 3569	MW939359.1	100	100	3.00E-79	Romania
PZ509194	B51	*Babesia canis*	18S rRNA	166	*Babesia canis* isolate 3569	MW939359.1	100	100	3.00E-79	Romania
PZ510385	C18	Coxiella-like endosymbiont	16S rRNA	1050	Coxiella-like endosymbiont isolate CLE	CP094229.1	100	99.9	0	South Africa
PZ510386	C25	Coxiella-like endosymbiont	16S rRNA	1050	Coxiella-like endosymbiont isolate CLE	CP094229.1	100	99.9	0	South Africa
PZ510387	C27	Coxiella-like endosymbiont	16S rRNA	1050	Coxiella-like endosymbiont isolate CLE	CP094229.1	100	99.9	0	South Africa
PZ510388	C44	Coxiella-like endosymbiont	16S rRNA	1050	Coxiella-like endosymbiont isolate CLE	CP094229.1	100	99.9	0	South Africa
PZ510389	C47	Coxiella-like endosymbiont	16S rRNA	1050	Coxiella-like endosymbiont isolate CLE	CP094229.1	100	99.9	0	South Africa
PZ510390	C48	Coxiella-like endosymbiont	16S rRNA	1050	Coxiella-like endosymbiont isolate CLE	CP094229.1	100	99.9	0	South Africa
PZ510391	C49	Coxiella-like endosymbiont	16S rRNA	1050	Coxiella-like endosymbiont isolate CLE	CP094229.1	100	99.9	0	South Africa
PZ510392	C50	Coxiella-like endosymbiont	16S rRNA	1050	Coxiella-like endosymbiont isolate CLE	CP094229.1	100	99.9	0	South Africa
PZ510393	C64	Coxiella-like endosymbiont	16S rRNA	1050	Coxiella-like endosymbiont isolate CLE	CP094229.1	100	99.9	0	South Africa
PZ510394	C65	Coxiella-like endosymbiont	16S rRNA	1050	Coxiella-like endosymbiont isolate CLE	CP094229.1	100	99.9	0	South Africa
PZ510395	C67	Coxiella-like endosymbiont	16S rRNA	1050	Coxiella-like endosymbiont isolate CLE	CP094229.1	100	99.9	0	South Africa

Accesion No.	Sample ID	Pathogen Detected	Target Gene	Sequence Length (bp)	Closest Match (BLASTn)	GenBank Accession No.	Query Cover (%)	Identity (%)	E-value	Country of Reference Sequence
PZ510396	C98	Coxiella-like endosymbiont	16S rRNA	1050	Coxiella-like endosymbiont isolate CLE	CP094229.1	100	99.52	0	South Africa
PZ510397	C99	Coxiella-like endosymbiont	16S rRNA	1050	Coxiella-like endosymbiont isolate CLE	CP094229.1	100	99.9	0	South Africa
PZ510398	C100	Coxiella-like endosymbiont	16S rRNA	1050	Coxiella-like endosymbiont isolate CLE	CP094229.1	100	99.9	0	South Africa
PZ510399	C101	Coxiella-like endosymbiont	16S rRNA	1050	Coxiella-like endosymbiont isolate CLE	CP094229.1	100	99.9	0	South Africa
PZ510400	C117	Coxiella-like endosymbiont	16S rRNA	1050	Coxiella-like endosymbiont isolate CLE	CP094229.1	100	99.62	0	South Africa
PZ510401	C118	Coxiella-like endosymbiont	16S rRNA	1050	Coxiella-like endosymbiont isolate CLE	CP094229.1	100	99.9	0	South Africa
PZ510402	C119	Coxiella-like endosymbiont	16S rRNA	1050	Coxiella-like endosymbiont isolate CLE	CP094229.1	100	99.9	0	South Africa
PZ510403	C120	Coxiella-like endosymbiont	16S rRNA	1050	Coxiella-like endosymbiont isolate CLE	CP094229.1	100	99.9	0	South Africa
PZ510404	C122	Coxiella-like endosymbiont	16S rRNA	1050	Coxiella-like endosymbiont isolate CLE	CP094229.1	100	99.9	0	South Africa
PZ510405	C132	Coxiella-like endosymbiont	16S rRNA	1050	Coxiella-like endosymbiont isolate CLE	CP094229.1	100	99.9	0	South Africa
PZ510406	C133	Coxiella-like endosymbiont	16S rRNA	1050	Coxiella-like endosymbiont isolate CLE	CP094229.1	100	99.7	0	South Africa
PZ510407	C142	Coxiella-like endosymbiont	16S rRNA	1050	Coxiella-like endosymbiont isolate CLE	CP094229.1	100	99.9	0	South Africa
PZ510408	C143	Coxiella-like endosymbiont	16S rRNA	1050	Coxiella-like endosymbiont isolate CLE	CP094229.1	100	99.9	0	South Africa
PZ510409	C161	Coxiella-like endosymbiont	16S rRNA	1050	Coxiella-like endosymbiont isolate CLE	CP094229.1	100	99.9	0	South Africa
PZ510410	C162	Coxiella-like endosymbiont	16S rRNA	1050	Coxiella-like endosymbiont isolate CLE	CP094229.1	100	99.9	0	South Africa
PZ510411	C163	Coxiella-like endosymbiont	16S rRNA	1050	Coxiella-like endosymbiont isolate CLE	CP094229.1	100	99.9	0	South Africa
PZ510412	C166	Coxiella-like endosymbiont	16S rRNA	1050	Coxiella-like endosymbiont isolate CLE	CP094229.1	100	99.9	0	South Africa
PZ510413	C167	Coxiella-like endosymbiont	16S rRNA	1050	Coxiella-like endosymbiont isolate CLE	CP094229.1	100	99.9	0	South Africa
PZ510414	C168	Coxiella-like endosymbiont	16S rRNA	713	Coxiella-like endosymbiont isolate CLE	CP094229.1	100	99.58	0	South Africa
PZ510415	C169	Coxiella-like endosymbiont	16S rRNA	1050	Coxiella-like endosymbiont isolate CLE	CP094229.1	100	99.9	0	South Africa
PZ510416	C170	Coxiella-like endosymbiont	16S rRNA	1050	Coxiella-like endosymbiont isolate CLE	CP094229.1	100	99.9	0	South Africa
PZ510417	C172	Coxiella-like endosymbiont	16S rRNA	1050	Coxiella-like endosymbiont isolate CLE	CP094229.1	100	99.9	0	South Africa
PZ510418	C183	Coxiella-like endosymbiont	16S rRNA	1050	Coxiella-like endosymbiont isolate CLE	CP094229.1	100	99.9	0	South Africa
PZ510419	C203	Coxiella-like endosymbiont	16S rRNA	1050	Coxiella-like endosymbiont isolate CLE	CP094229.1	100	99.9	0	South Africa
PZ510420	C204	Coxiella-like endosymbiont	16S rRNA	1050	Coxiella-like endosymbiont isolate CLE	CP094229.1	100	99.9	0	South Africa
PZ510421	C205	Coxiella-like endosymbiont	16S rRNA	1050	Coxiella-like endosymbiont isolate CLE	CP094229.1	100	99.9	0	South Africa
PZ510422	C206	Coxiella-like endosymbiont	16S rRNA	1050	Coxiella-like endosymbiont isolate CLE	CP094229.1	100	99.9	0	South Africa
PZ510423	C232	Coxiella-like endosymbiont	16S rRNA	1050	Coxiella-like endosymbiont isolate CLE	CP094229.1	100	99.9	0	South Africa
PZ510424	C233	Coxiella-like endosymbiont	16S rRNA	1050	Coxiella-like endosymbiont isolate CLE	CP094229.1	100	99.9	0	South Africa
PZ510425	C241	Coxiella-like endosymbiont	16S rRNA	1050	Coxiella-like endosymbiont isolate CLE	CP094229.1	100	99.9	0	South Africa
PZ510426	C243	Coxiella-like endosymbiont	16S rRNA	1050	Coxiella-like endosymbiont isolate CLE	CP094229.1	100	99.9	0	South Africa
PZ510427	C244	Coxiella-like endosymbiont	16S rRNA	1007	Coxiella-like endosymbiont isolate CLE	CP094229.1	100	99.9	0	South Africa
PZ510428	C245	Coxiella-like endosymbiont	16S rRNA	1050	Coxiella-like endosymbiont isolate CLE	CP094229.1	100	99.9	0	South Africa
PZ510429	C269	Coxiella-like endosymbiont	16S rRNA	1050	Coxiella-like endosymbiont isolate CLE	CP094229.1	100	99.9	0	South Africa
PZ510430	C270	Coxiella-like endosymbiont	16S rRNA	1050	Coxiella-like endosymbiont isolate CLE	CP094229.1	100	99.9	0	South Africa
PZ510431	C271	Coxiella-like endosymbiont	16S rRNA	1050	Coxiella-like endosymbiont isolate CLE	CP094229.1	100	99.71	0	South Africa
PZ510432	C272	Coxiella-like endosymbiont	16S rRNA	1050	Coxiella-like endosymbiont isolate CLE	CP094229.1	100	99.9	0	South Africa
PZ510433	C273	Coxiella-like endosymbiont	16S rRNA	1050	Coxiella-like endosymbiont isolate CLE	CP094229.1	100	99.9	0	South Africa
PZ510434	C275	Coxiella-like endosymbiont	16S rRNA	1050	Coxiella-like endosymbiont isolate CLE	CP094229.1	100	99.81	0	South Africa
PZ510435	C276	Coxiella-like endosymbiont	16S rRNA	1050	Coxiella-like endosymbiont isolate CLE	CP094229.1	100	99.9	0	South Africa
PZ510436	C288	Coxiella-like endosymbiont	16S rRNA	1050	Coxiella-like endosymbiont isolate CLE	CP094229.1	100	99.9	0	South Africa
PZ510437	C289	Coxiella-like endosymbiont	16S rRNA	1050	Coxiella-like endosymbiont isolate CLE	CP094229.1	100	99.9	0	South Africa
PZ510438	C290	Coxiella-like endosymbiont	16S rRNA	1050	Coxiella-like endosymbiont isolate CLE	CP094229.1	100	99.9	0	South Africa
PZ510439	C304	Coxiella-like endosymbiont	16S rRNA	1050	Coxiella-like endosymbiont isolate CLE	CP094229.1	100	99.9	0	South Africa
